# Use of viral load surveillance data to assess linkage to care for persons with HIV released from corrections

**DOI:** 10.1371/journal.pone.0192074

**Published:** 2018-02-12

**Authors:** Brian T. Montague, Betsey John, Cara Sammartino, Michael Costa, Dawn Fukuda, Liza Solomon, Josiah D. Rich

**Affiliations:** 1 Department of Medicine, Division of Infectious Diseases, University of Colorado Denver School of Medicine, Aurora, Colorado, United States of America; 2 Bureau of Infectious Disease and Laboratory Science, Massachusetts Department of Public Health, Boston, Massachusetts, United States of America; 3 Department of Health Sciences, Johnson and Wales University, Providence, Rhode Island, United States of America; 4 Abt Associates, Cambridge, Massachusetts, United States of America; 5 Miriam Hospital, Providence, Rhode Island, United States of America; 6 Department of Medicine, Division of Infectious Diseases, Warren Alpert School of Medicine at Brown University, Providence, Rhode Island, United States of America; National and Kapodistrian University of Athens, GREECE

## Abstract

Incarcerated people remain a priority group in efforts to control and reverse the HIV epidemic. Following release, social instability and reengagement in key transmission risk behaviors increase the risk of secondary transmission of HIV. Targeted programs have been developed to facilitate reengagement in care on reentry. Evaluation of the impact of these initiatives requires a systematic, confidential, framework for assessment of linkage to care for persons released from corrections. By linking HIV viral load surveillance data to corrections release data, the time to the first laboratory monitoring service in the community as well as the virologic status can be assessed. Using this method, we linked release data for sentenced individuals released from Massachusetts state correctional facilities in 2012 to HIV surveillance data from the Massachusetts HIV/AIDS Surveillance Program (MHASP) for the years 2012–2013. We identified 41 individuals with HIV released in 2012. Ninety-one percent had identified virologic assessments post release, 41% within 30 days. Thirty-three percent did not have a viral load assessed for more than 90 days and 31% had detectable virus at the time of their initial assessment. Persons with longer incarcerations (> 180 days) were more likely to have suppressed viral load at the time of follow-up (p = 0.05). This work demonstrates the important value of HIV laboratory surveillance data and correctional release data as a tool to assess linkage to care following release from corrections. We encourage jurisdictions to explore utilizing similar methodology to evaluate the effectiveness of the linkage to HIV care after release from incarceration.

## Introduction

Incarcerated people remain a priority group in efforts to control and ultimately reverse the HIV epidemic.[1] The Bureau of Justice Statistics estimates case rate among persons in corrections, as of 2010, at 146 cases per 10,000 inmates. It has further been estimated previously that 14% of persons with HIV pass through the corrections system annually.[2] Persons with HIV who are incarcerated may be undiagnosed or out of care and off treatment, making periods of incarceration important times to identify these individuals and reconnect them to needed services.[3]

The period before and after incarceration has been shown to be a high-risk time for HIV transmission.[4–7] In the time leading up to incarceration, persons may experience significant social instability leading to gaps in care and in treatment with an estimated 42% and 78% engaged in care and only 1% and 35%[3] with viral suppression on treatment. Following release, social instability and reengagement in key transmission risk behaviors increase the risk of secondary transmission of HIV. Estimates of engagement in care during this time have varied widely from as low as 28% to as high as 96% in some model programs with most surveys showing proportions engaged less than 75%. There is less data regarding the proportions virologically suppressed at follow-up, but a prior survey from CT showed 61% virologically suppressed 12 weeks following release from prison.[8] A multisite study of jail populations showed only 26% virologically suppressed 6 months following release from jail.[9]

Targeted programs have been developed to complement corrections release planning as a means of facilitating reengagement in care for persons with HIV on reentry.[10–12] Evaluation of the impact of these initiatives requires a systematic, confidential, framework for assessment of linkage to care for persons released from corrections.[13] We developed such a framework based on the linkage of corrections release data from the National Corrections Reporting Program and clinical service data from Ryan White funded care providers in the form of the Ryan White client level data reporting.[14, 15] While in many jurisdictions, Ryan White funded service providers are key safety net providers serving people in the post-release period, Medicaid expansion and efforts to enroll patients in Medicaid prerelease may lead to a larger portion of services being provided by non-Ryan White funded care providers. [16–20] The Ryan White reporting files in these jurisdictions may come to include a smaller portion of the services provided to persons with HIV on reentry.

State and CDC viral load surveillance data offer a source of clinical data that is independent of patients’ insurance status. Given that, in most cases, the viral load will be assessed at intake to a clinic, the time to the first viral load assessment in the community as assessed through electronic laboratory reporting (ELR) data may provide an important indicator of the timeliness of linkage to care on release from corrections. Consideration of the use of viral load surveillance for assessing linkage to care and retention in care is just beginning and perceptions of confidentiality concerns and policy barriers may limit its use in many jurisdictions.[21–24] This study is the first use of surveillance viral load and CD4 data and corrections release data to measure effectiveness of linkage to HIV care after release from incarceration.

## Methods

Data for sentenced persons released from Massachusetts state correctional facilities formatted for the National Corrections Reporting Program and HIV surveillance data from the Massachusetts HIV/AIDS Surveillance Program (MHASP) were obtained for the years 2012 and 2013. The corrections release dataset included data for all releasees regardless of HIV status and included personal identifiers for linkage as well as the incarceration date and release date. HIV surveillance data included records for all individuals with known HIV diagnosed and reported to the MHASP. The records included identifiers for linkage together with the dates and results for all reported HIV viral load and CD4+ T-cell count assessments in 2012 and 2013.

Data linkage occurred by employing the algorithm used by the Health Resources and Services Administration (HRSA), HIV/AIDS Bureau (HAB) for its Ryan White Services Report (RSR) client-level data. The algorithm creates an encrypted Unique Client Identifier (eUCI) derived from the first and third characters of the first and last name, the full date of birth, and the gender. The hashing algorithm used for encryption is a trap door algorithm that prevents recovery of the source data.[25] This method performed comparably to probabilistic matching in validation studies and has been previously used to assess linkage to care using the corrections release data and client level data reporting from Ryan White funded care providers in Rhode Island and North Carolina.[14, 15]

The corrections data file was transferred to Abt Associates where eUCIs were generated for all names and aliases and the identifiers were removed. An analysis file with eUCIs, incarceration dates and release dates was transferred to the MHASP for linkage. Data linkage between the corrections records and the HIV/AIDS surveillance data was conducted onsite at the Massachusetts Department of Public Health (MDPH) by epidemiologists from the MHASP using Statistical Analysis System(SAS) using an algorithm developed at Abt Associates for prior similar linkage studies.[13, 15] This team was trained in the use of the algorithm to assure fidelity of the matching process. Following record linkage, the eUCIs were replaced with arbitrary identifiers and the dates were masked through addition of a random integer known only to the epidemiologist within MDPH. The generated fully de-identified data set was then transferred from MDPH to the study team for analysis.

The linked data records were summarized by key demographic, clinical- and incarceration-related factors as well as the distribution of viral load and CD4+ T-cell assessments by individual. Duration of incarceration was categorized as less than 180 days or greater than 180 days. CD4+ T-cell results were categorized as: less than 200, 200–500 and greater than 500. Persons with multiple CD4+ T-cell tests were assessed as stable, increasing or decreasing across the measurement period. Records with CD4 percentage without a CD4+ T-cell count were excluded for considerations of CD4 status. The outcomes considered include time to the first HIV viral load assessment in the community after release and virologic status (suppressed or not suppressed) at the first assessment.

Kaplan Meier curves were used to depict the distribution of time to first virologic assessment. All individuals without identified viral load assessments were censored as of the time of the last documented viral load assessment in the dataset which was considered the end of the follow-up period. The log-rank test was used to assess for differences in time to first assessment by key demographic factors including gender, race/ethnicity, HIV exposure mode and duration of incarceration. Differences in virologic status at linkage by the same factors were assessed using Fischer’s exact tests. All statistical analyses were performed using Stata/SE version 14.0.

This protocol was reviewed and approved by the Institutional Review Boards within the Massachusetts Departments of Public Health and Massachusetts Department of Corrections, Abt Associates, the Miriam Hospital and the Office for Human Research Protections.

## Results

A total of 9,657 releases were documented in the NCRP data file. A total of 56 matches were identified between the NCRP data file and the Massachusetts HIV/AIDS surveillance data set. Of these 51 had identified assessments in the community post-release. Forty-eight had multiple assessments. Demographic data for the matched sample are presented in Table 1. Seventy percent were men. Twenty-eight percent were identified as black and 36% were identified as Hispanic. Seventy four percent reported injection drug use (IDU) as part of their exposure mode for HIV acquisition with 63% identifying IDU alone and 11% were reported male to male sexual contact (MSM) and IDU. Two percent were identified as deceased. The median time of incarceration was 474 days with interquartile range of 1115 days (128–1243 days). The median follow-up time for persons released was 490 days with an interquartile range of 207 days.

**Table 1:** Demographic and incarceration related factors for persons with HIV released from Massachusetts state correctional facilities in 2012.

	n	%
Gender		
Male	39	70
Female	17	30
Race/Ethnicity		
White (non-Hispanic/Latino)	20	36
Black (non-Hispanic/Latino)	16	28
Hispanic/Latino	20	36
Age Group		
25–34	5	9
35–44	15	27
45–54	20	36
> 55–64	16	29
HIV Exposure Mode		
Heterosexual	8	14
IDU	35	63
MSM	<5	
MSM/IDU	6	11
Other	<5	
Status		
Not Deceased	55	98
Deceased	1	2

Of the 56 matches, 51 (91%) had identified HIV viral load assessments post-release. Of these 94% had their first virologic assessment within 365 days. Eighty-six percent had more than one virologic assessment in the post-release period. A single individual had an identified CD4 assessment post-release without a viral load assessment. Viral load data during the period of incarceration was available for only 3 out of 56 (5%) whereas CD4 data from during the period of incarceration was available for 33 (56%). The median CD4 count was 330 with interquartile range 421 (123–544). Of the 33 with laboratory assessment in corrections, 27 had follow-up CD4 assessments post-release. Ninety-six percent of these had stable or improved CD4+ T-cell count at the time of assessment post release (see Table 2).

**Table 2:** Change in CD4 for those who had CD4 count data while in prison and then at a follow up post release.

CD4	No Follow Up	Follow Up Decline	Follow Up Stable	Follow Up Increased
<200	-	0 (0)	4(100)	0 (0)
200–500	2*	0 (0)	9(56)	7 (44)
>500	4*	1 (14)	6 (86)	0 (0)
Overall	6	1 (4)	19 (70)	7 (26)

* includes 1 individual with CD4 percentage post release without an accompanying CD4 count

**Fig 1:**
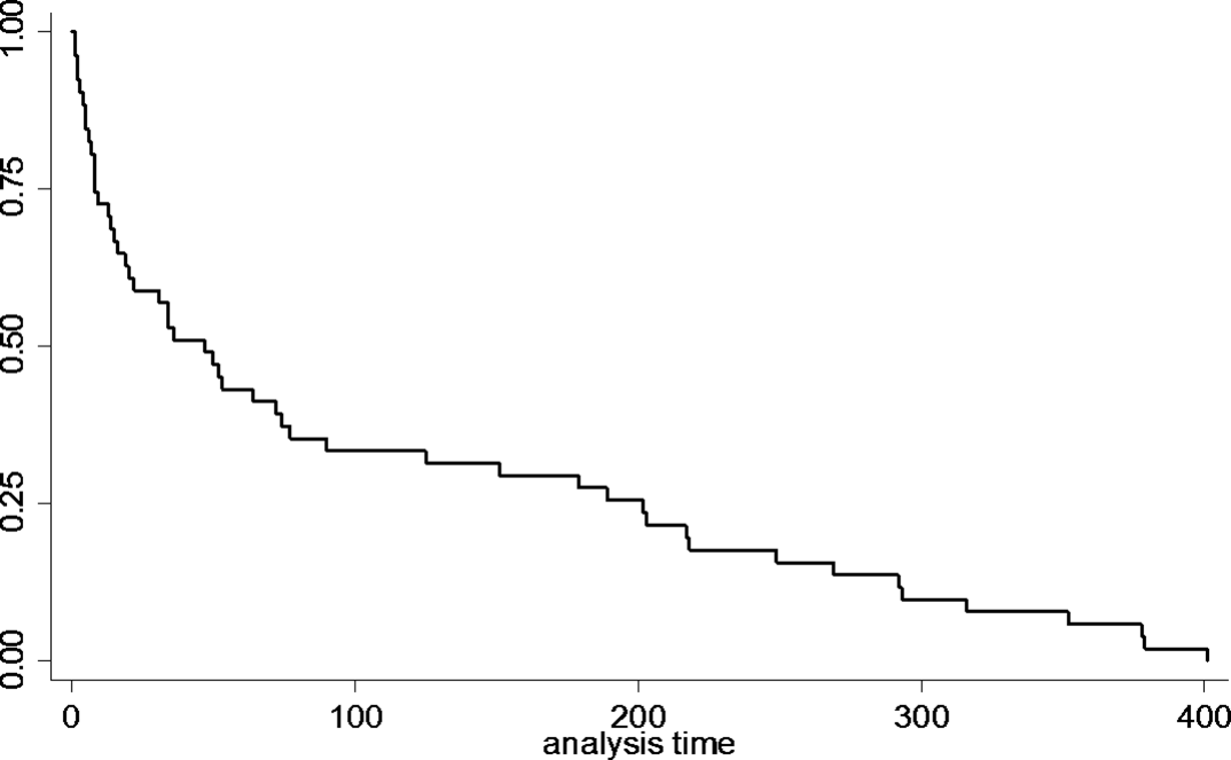
Massachusetts state corrections release cohort and matches to HIV viral surveillance data for the 2012.

Forty-one percent overall had their first viral load assessment within 30 days of release. The distribution of time to first service is presented in Fig 1. No significant differences in time to first service were identified by gender, race/ethnicity, HIV transmission risk, or duration of incarceration. Thirty-three percent did not have a viral load assessed for more than 90 days and 31% had detectable virus at the time of their initial assessment (see Table 3). Persons with longer incarcerations (> 180 days) were more likely to have suppressed viral load at the time of follow-up (p = 0.05). No significant differences were found in time to first laboratory assessment or proportion with suppressed viral load at first assessment post-release by race/ethnicity or exposure mode. Women on average linked later than men, although the small number of women in the sample limited the ability to establish statistically significant differences by gender.

**Table 3:** Factors associated with viral load at first service.

	Viral Load			
	<200	200–1000	>1000	Fisher’s Exact
	n (%)	n (%)	n (%)	
Gender				
Male	26 (74.3)	2 (5.7)	7 (20)	0.21
Female	8 (50)	2 (12.5)	6 (37.5)	
Race/Ethnicity				
White (non-hispanic)	13 (68.4)	2 (10.5)	4 (21.1)	0.75
Black (non-hispanic)	7 (53.8)	1 (7.7)	5 (38.5)	
Hispanic	14 (73.7)	1 (5.3)	4 (21)	
HIV Risk Factor				
Hetero Contact	4 (50)	2 (25)	2 (25)	0.66
IDU	21 (67.7)	2 (6.5)	8 (25.8)	
MSM	2 (100)	0 (0)	0 (0)	
MSM/IDU	5 (83.3)	0 (0)	1 (16.7)	
Other	2 (50)	0 (0)	2 (50)	
Time Incarcerated				
<180 days	**7 (46.7)**	**3 (20)**	**5 (33.3)**	**0.04**
More than 180 days	**27 (75)**	**1 (2.8)**	**8 (22.2)**	
Time to Care				
<30 days	14 (66.7)	3 (14.3)	4 (19)	0.30
30–90 days	10 (76.9)	1 (7.7)	2 (15.4)	
>90 days	10 (58.8)	0 (0)	7 (41.2)	

## Discussion

This work demonstrates the important value of HIV laboratory surveillance data and correctional release data as a tool to assess linkage to care following release from corrections. By linking corrections release data and laboratory surveillance data, we identified 56 individuals with HIV released from a state correctional facility in Massachusetts of whom 91% were identified to have linked to care in the community, as indicated by receipt of a viral load test following release, during 2012 or 2013. Among those who linked to care in the community only 41% did so within 30 days. This is typically the maximum duration of medication provided to persons on release. Detectable viremia was present in 31% of persons assessed, indicating significant gaps in treatment post-release. Notably, however, among those with delays in their first laboratory test greater than 90 days, 59% were suppressed at the time of their first assessment suggesting either that there is incomplete service capture in the surveillance files or patients may have had access to care including medications without viral testing. This can be compared to the overall estimated rate of virologic suppression for persons with HIV in MA of 65% and indicates significant gaps in treatment in the post release period.[26] All individuals were followed for at least one year which was sufficient time to identify linkage for 94% of those who ultimately linked. It is possible that a portion of the 9% not demonstrated to link in 2012 or 2013 may have followed up for care in 2014.

Prior analyses of linkage to care using Ryan White data have suggested that longer periods of incarceration, greater than 180 days, are associated with earlier linkage to care.[15] In this analysis we found that persons incarcerated for less than 180 days were more likely to have detectable virus at their first assessment in the community though there was no difference in time to linkage. Incarceration provides an opportunity for patients to engage in care, including for some the ability to access ancillary mental health or other support services. This ability may be limited in the context of shorter incarcerations. In addition, persons out of care at the time of incarceration may be less likely to achieve HIV viral suppression while incarcerated if the duration of incarceration is short. Given the lack of viral load assessments during incarceration, it was not possible to assess whether or not those with detectable virus were trending down and the observed values were consistent with ongoing antiretroviral therapy.

Using this framework, it is not possible to establish whether an individual’s HIV status was known at the time of incarceration except in cases for which testing was performed during the period of incarceration. In a serosurvey of persons incarcerated in New York City jails in 2006, 13% of individuals identified as HIV positive on the survey had previously reported their status.[27] Notably 25% of individuals were not tested and not known to have been previously diagnosed. Persons of unknown status while incarcerated will be unable to access targeted services to support linkage to care. A viral load test performed in the community following release, could equally represent a diagnostic test performed for persons with concern for acute HIV. Based on previously observed low rates of new HIV diagnoses identified through routine testing in probation and parole, if this occurs, it would likely be a small component of the overall sample.[28] In most states, laboratory surveillance data is coupled with name- based reporting for new diagnoses of HIV and using those records, new diagnoses following release could be specifically identified. There we no instances of this in our sample.

HIV laboratory surveillance data offers significant potential advantages over either practice-based or clinical payer-based data sets. HIV laboratory surveillance datasets are becoming increasingly well developed around the country.[29] In this sample, there remain some indications of incomplete data particularly in the CD4+ T-cell reporting and in the reporting of data for labs collected during the period of incarceration. Though release of this data to outside parties may pose confidentiality concerns, this analysis demonstrates that the data may be confidentially matched as part of program monitoring through effective collaboration between state and federal agencies.

## Limitations

This analysis is limited to individuals diagnosed and reported to the HIV surveillance system. For the 9% with no identified viral load testing post release, it is possible that a portion of these linked to care outside of Massachusetts. The 56 individuals can be compared to the estimated population of HIV+ individuals who reside in correctional facilities, which has trended down from 264 in 2008 to 206 in 2010.[30] The 56 HIV+ individuals identified likely encompass a significant portion of those with HIV released during the year. Though this work does not describe the experience of the 9% who do not link to care, the experience of those who ultimately link is likely informative with regard to both facilitators of and barriers to linkage. The information obtained can support development of programs that may equally benefit those currently being lost to care.

The development of systems to ensure the completeness and accuracy of HIV and electronic laboratory surveillance (ELR) data is key to its value for clinical care monitoring. 2012 was the first year in Massachusetts when the full laboratory reporting was implemented and considered complete. The pattern of observed lab monitoring in corrections, specifically individuals for whom CD4+ T-cell testing was performed but no viral load tests were reported, suggest that some gaps may remain. This may be due to the fact that many of the correctional facilities use an external out of state commercial lab that report via paper which caused omission of their laboratory results from the electronic surveillance data. A Massachusetts Department of Health audit of HIV care performed in Massachusetts correctional facilities for the period of August through September notably found that at least one viral load assessment was available for 99 percent of incarcerated persons with HIV and 84% were virologically suppressed.[31] Nationally surveillance programs are continually developing. As states work to improve the integrity and completeness of these reporting systems, the value of ELR/HIV surveillance data as a clinical monitoring tool will continue to rise. Extension of this framework to use with national aggregated data would both provide important information nationally regarding linkage to care on reentry and reduce the concerns for apparent false negative linkage to services received out of state.

Persons released from corrections are at risk for both interruptions in care and poor retention in care. Though this analysis focused on persons released from prison, the value may be even greater when applied to persons released from jails given that they release approximately 10 times the number of persons with HIV in a given year. Longer-term follow-up would be needed to assess patterns of retention in care in this population. Since regular viral load monitoring is standard in the context of HIV care visits, viral load surveillance data would provide an important tool for assessing retention in care and effectiveness of care over time.

## Conclusions

A major gap in our system of care for people with HIV occurs at the time of release from incarceration.[32, 33] In order to address this gap, we need a reliable measure of linkage to care following release from incarceration. We describe a system that can be easily replicated in any jurisdiction that has access to correctional data and viral load surveillance data, as well as the ability to analyze the data in a confidential way. This can be used to identify areas of need, and tracked to assess the effectiveness of linkage interventions over time. Routine monitoring of linkage to care, retention in care, and viral suppression following release from corrections would enable an accurate tracking of outcomes across the HIV Care Continuum for this National HIV/AIDS Strategy (NHAS) priority population. [1] We encourage jurisdictions to explore utilizing similar methodology to evaluate the effectiveness of the linkage to HIV care after release from incarceration.

## References

[1] Policy OoNA. National HIV/AIDS Strategy for the United States: Office of National AIDS Policy; 2010 [2/2/2011]. Available from: http://www.whitehouse.gov/administration/eop/onap/nhas/.

[2] Spaulding AC, Seals RM, Page MJ, Brzozowski AK, Rhodes W, Hammett TM. HIV/AIDS among inmates of and releasees from US correctional facilities, 2006: declining share of epidemic but persistent public health opportunity. PLoS One. 2009;4(11):e7558 doi: 10.1371/journal.pone.0007558 ; PubMed Central PMCID: PMC2771281. 19907649 10.1371/journal.pone.0007558 PMC2771281

[3] Iroh PA, Mayo H, Nijhawan AE. The HIV Care Cascade Before, During, and After Incarceration: A Systematic Review and Data Synthesis. Am J Public Health. 2015;105(7):e5–16. doi: 10.2105/AJPH.2015.302635 ; PubMed Central PMCID: PMCPMC4463395. 25973818 10.2105/AJPH.2015.302635 PMC4463395

[4] Khan MR, Miller WC, Schoenbach VJ, Weir SS, Kaufman JS, Wohl DA, et al Timing and duration of incarceration and high-risk sexual partnerships among African Americans in North Carolina. Ann Epidemiol. 2008;18(5):403–10. Epub 2008/04/09. doi: S1047-2797(08)00004-5 [pii] doi: 10.1016/j.annepidem.2007.12.003 ; PubMed Central PMCID: PMC2877367. 18395464 10.1016/j.annepidem.2007.12.003 PMC2877367

[5] Khan MR, Wohl DA, Weir SS, Adimora AA, Moseley C, Norcott K, et al Incarceration and risky sexual partnerships in a southern US city. J Urban Health. 2008;85(1):100–13. Epub 2007/11/21. doi: 10.1007/s11524-007-9237-8 ; PubMed Central PMCID: PMC2430135. 18027088 10.1007/s11524-007-9237-8 PMC2430135

[6] Adams LM, Kendall S, Smith A, Quigley E, Stuewig JB, Tangney JP. HIV risk behaviors of male and female jail inmates prior to incarceration and one year post-release. AIDS Behav. 2013;17(8):2685–94. doi: 10.1007/s10461-011-9990-2 ; PubMed Central PMCID: PMCPMC4293014. 21779954 10.1007/s10461-011-9990-2 PMC4293014

[7] Kouyoumdjian FG, Calzavara LM, Kiefer L, Main C, Bondy SJ. Drug use prior to incarceration and associated socio-behavioural factors among males in a provincial correctional facility in Ontario, Canada. Can J Public Health. 2014;105(3):e198–202. . 25165839 10.17269/cjph.105.4193 PMC6972117

[8] Springer SA, Chen S, Altice FL. Improved HIV and substance abuse treatment outcomes for released HIV-infected prisoners: the impact of buprenorphine treatment. J Urban Health. 2010;87(4):592–602. doi: 10.1007/s11524-010-9438-4 ; PubMed Central PMCID: PMCPMC2900572. 20177974 10.1007/s11524-010-9438-4 PMC2900572

[9] Spaulding AC, Messina LC, Kim BI, Chung KW, Lincoln T, Teixeira P, et al Planning for success predicts virus suppressed: results of a non-controlled, observational study of factors associated with viral suppression among HIV-positive persons following jail release. AIDS Behav. 2013;17 Suppl 2:S203–11. doi: 10.1007/s10461-012-0341-8 . 23076719 10.1007/s10461-012-0341-8

[10] Booker CA, Flygare CT, Solomon L, Ball SW, Pustell MR, Bazerman LB, et al Linkage to HIV care for jail detainees: findings from detention to the first 30 days after release. AIDS Behav. 2013;17 Suppl 2:S128–36. doi: 10.1007/s10461-012-0354-3 . 23224290 10.1007/s10461-012-0354-3

[11] Zaller ND, Holmes L, Dyl AC, Mitty JA, Beckwith CG, Flanigan TP, et al Linkage to treatment and supportive services among HIV-positive ex-offenders in Project Bridge. J Health Care Poor Underserved. 2008;19(2):522–31. doi: 10.1353/hpu.0.0030 . 18469423 10.1353/hpu.0.0030

[12] Wohl DA, Scheyett A, Golin CE, White B, Matuszewski J, Bowling M, et al Intensive case management before and after prison release is no more effective than comprehensive pre-release discharge planning in linking HIV-infected prisoners to care: a randomized trial. AIDS Behav. 2011;15(2):356–64. doi: 10.1007/s10461-010-9843-4 ; PubMed Central PMCID: PMC3532052. 21042930 10.1007/s10461-010-9843-4 PMC3532052

[13] Montague BT, Rosen DL, Solomon L, Nunn A, Green T, Costa M, et al Tracking linkage to HIV care for former prisoners: a public health priority. Virulence. 2012;3(3):319–24. doi: 10.4161/viru.20432 ; PubMed Central PMCID: PMC3442844. 22561157 10.4161/viru.20432 PMC3442844

[14] Gutman R, Sammartino CJ, Green TC, Montague BT. Error adjustments for file linking methods using encrypted unique client identifier (eUCI) with application to recently released prisoners who are HIV. Stat Med. 2015 doi: 10.1002/sim.6586 . 26202853 10.1002/sim.6586 PMC4715569

[15] Montague BT, Rosen D, Sammartino CJ, Costa M, Gutman R, Solomon L, et al Systematic Assessment of Linkage to Care for Persons with HIV Released from Corrections Facilities Using Existing Datasets AIDS Pt Care & STDs. 2016;publication pending. 10.1089/apc.2015.0258 PMC4753628 26836237

[16] Bandara SN, Huskamp HA, Riedel LE, McGinty EE, Webster D, Toone RE, et al Leveraging The Affordable Care Act To Enroll Justice-Involved Populations In Medicaid: State And Local Efforts. Health Aff (Millwood). 2015;34(12):2044–51. doi: 10.1377/hlthaff.2015.0668 ; PubMed Central PMCID: PMCPMC4880991. 26643624 10.1377/hlthaff.2015.0668 PMC4880991

[17] Boutwell AE, Freedman J. Coverage expansion and the criminal justice-involved population: implications for plans and service connectivity. Health Aff (Millwood). 2014;33(3):482–6. doi: 10.1377/hlthaff.2013.1131 . 24590949 10.1377/hlthaff.2013.1131

[18] Riedel LE, Barry CL, McGinty EE, Bandara SN, Webster DW, Toone RE, et al Improving Health Care Linkages for Persons: The Cook County Jail Medicaid Enrollment Initiative. J Correct Health Care. 2016;22(3):189–99. doi: 10.1177/1078345816653199 . 27302704 10.1177/1078345816653199

[19] Rosen DL, Grodensky CA, Holley TK. Federally-Assisted Healthcare Coverage among Male State Prisoners with Chronic Health Problems. PLoS One. 2016;11(8):e0160085 doi: 10.1371/journal.pone.0160085 . 27479089 10.1371/journal.pone.0160085 PMC4968827

[20] Rosen DL, Dumont DM, Cislo AM, Brockmann BW, Traver A, Rich JD. Medicaid policies and practices in US state prison systems. Am J Public Health. 2014;104(3):418–20. doi: 10.2105/AJPH.2013.301563 ; PubMed Central PMCID: PMCPMC3953759. 24432881 10.2105/AJPH.2013.301563 PMC3953759

[21] Braunstein SL, Robertson MM, Myers J, Nash D. Using HIV Viral Load from Surveillance to Estimate the Timing of Antiretroviral Therapy Initiation. JAIDS. 2016. Epub 5/5/2016. 10.1097/QAI.0000000000001052 PMC5584788 27152466

[22] Sabharwal CJ, Braunstein SL, Robbins RS, Shepard CW. Optimizing the use of surveillance data for monitoring the care status of persons recently diagnosed with HIV in NYC. J Acquir Immune Defic Syndr. 2014;65(5):571–8. doi: 10.1097/QAI.0000000000000077 . 24326601 10.1097/QAI.0000000000000077

[23] Terzian AS, Bodach SD, Wiewel EW, Sepkowitz K, Bernard MA, Braunstein SL, et al Novel use of surveillance data to detect HIV-infected persons with sustained high viral load and durable virologic suppression in New York City. PLoS One. 2012;7(1):e29679 doi: 10.1371/journal.pone.0029679 ; PubMed Central PMCID: PMCPMC3265470. 22291892 10.1371/journal.pone.0029679 PMC3265470

[24] Wiewel EW, Braunstein SL, Xia Q, Shepard CW, Torian LV. Monitoring outcomes for newly diagnosed and prevalent HIV cases using a care continuum created with New York city surveillance data. J Acquir Immune Defic Syndr. 2015;68(2):217–26. doi: 10.1097/QAI.0000000000000424 . 25394192 10.1097/QAI.0000000000000424

[25] HRSA HIV/AIDS Bureau DaRTT. Encrypted Unique Client Identifier (eUCI): Application and User Guide: Target Center; [8/9/2016]. Available from: https://careacttarget.org/library/encrypted-unique-client-identifier-euci-application-and-user-guide.

[26] Services MDoHaH. Massachusetts HIV Care Continuum Fact Sheet [61/2007]. Available from: http://www.mass.gov/eohhs/docs/dph/aids/2016-profiles/hiv-care-continuum-factsheet.pdf.

[27] Begier EM, Bennani Y, Forgione L, Punsalang A, Hanna DB, Herrera J, et al Undiagnosed HIV infection among New York City jail entrants, 2006: results of a blinded serosurvey. J Acquir Immune Defic Syndr. 2010;54(1):93–101. doi: 10.1097/QAI.0b013e3181c98fa8 . 20042868 10.1097/QAI.0b013e3181c98fa8

[28] Gordon MS, Kinlock TW, McKenzie M, Wilson ME, Rich JD. Rapid HIV testing for individuals on probation/parole: outcomes of an intervention trial. AIDS Behav. 2013;17(6):2022–30. doi: 10.1007/s10461-013-0456-6 ; PubMed Central PMCID: PMCPMC3674156. 23536140 10.1007/s10461-013-0456-6 PMC3674156

[29] CDC. State Laboratory Reporting Laws: Viral Load and CD4 Requirements 2016 [12/28/2016]. Available from: https://www.cdc.gov/hiv/policies/law/states/reporting.html

[30] Maruschak L. HIV In Prisons, 2001–2010. BJS, 2012.

[31] Marco A, Esteban JI, Sole C, da Silva A, Ortiz J, Roget M, et al Hepatitis C virus reinfection among prisoners with sustained virological response after treatment for chronic hepatitis C. J Hepatol. 2013;59(1):45–51. doi: 10.1016/j.jhep.2013.03.008 . 23523577 10.1016/j.jhep.2013.03.008

[32] Baillargeon J, Giordano TP, Rich JD, Wu ZH, Wells K, Pollock BH, et al Accessing antiretroviral therapy following release from prison. JAMA. 2009;301(8):848–57. doi: 10.1001/jama.2009.202 ; PubMed Central PMCID: PMC2936238. 19244192 10.1001/jama.2009.202 PMC2936238

[33] Baillargeon JG, Giordano TP, Harzke AJ, Baillargeon G, Rich JD, Paar DP. Enrollment in outpatient care among newly released prison inmates with HIV infection. Public Health Rep. 2010;125 Suppl 1:64–71. doi: 10.1177/00333549101250S109 ; PubMed Central PMCID: PMC2788410. 20408389 10.1177/00333549101250S109 PMC2788410

